# GluA1 AMPAR subunit deletion reduces the hedonic response to sucrose but leaves satiety and conditioned responses intact

**DOI:** 10.1038/s41598-017-07542-9

**Published:** 2017-08-07

**Authors:** Joseph M. Austen, Rolf Sprengel, David J. Sanderson

**Affiliations:** 1 Department of Psychology, Durham University, Science Site, South Road, Durham, DH1 3LE UK; 2 Max Planck Research Group at the Inst. for Anatomy and Cell Biology, Heidelberg University, D69120 Heidelberg, Germany

## Abstract

The GluA1 subunit of the AMPA receptor has been implicated in schizophrenia. While GluA1 is important for cognition, it is not clear what the role of GluA1 is in hedonic responses that are relevant to the negative symptoms of disorders such as schizophrenia. Here, we tested mice that lack GluA1 (*Gria1* ^−/−^ mice) on consumption of sucrose solutions using a licking microstructure analysis. GluA1 deletion drastically reduced palatability (as measured by the mean lick cluster size) across a range of sucrose concentrations. Although initial lick rates were reduced, measures of consumption across long periods of access to sucrose solutions were not affected by GluA1 deletion and *Gria1* ^−/−^ mice showed normal satiety responses to high sucrose concentrations. GluA1 deletion also failed to impair flavour conditioning, in which increased intake of a flavour occurred as a consequence of prior pairing with a high sucrose concentration. These results demonstrate that GluA1 plays a role in responding on the basis of palatability rather than other properties, such as the automatic and learnt post-ingestive, nutritional consequences of sucrose. Therefore, *Gria1* ^−/−^ mice provide a potential model of anhedonia, adding converging evidence to the role of glutamatergic dysfunction in various symptoms of schizophrenia and related disorders.

## Introduction

Glutamatergic dysfunction has been proposed as a potential cause of schizophrenia^1^. Recently the *Gria1* gene that encodes for the GluA1 subunit of the AMPA receptor for glutamate has been found to show genome wide association to schizophrenia^2, 3^. Furthermore, post-mortem tests have revealed a reduction in hippocampal GluA1 mRNA^4, 5^, and GluA1^6^ and AMPA binding sites^7^ in schizophrenia patients. Genetically modified mice that lack a functional *Gria1* gene now provide a useful means of studying the causal role of glutamatergic dysfunction in neuropsychiatric disorders.

Knockout of *Gria1* in mice (*Gria1* ^−/−^ mice) results in impaired hippocampal plasticity^8–11^ and causes cognitive deficits that are associated with dysfunction of the hippocampus^12–14^ and amygdala^15, 16^. Furthermore, *Gria1* ^−/−^ mice show behavioural abnormalities that are relevant to schizophrenia suggesting that *Gria1* deletion models components of the disorder^17–19^. In particular *Gria1* ^*−/−*^ mice fail to reduce attention to recently experienced stimuli as a consequence of impaired short-term habituation^14, 20–22^, suggesting that GluA1 deletion may model aspects of aberrant salience in disorders such as schizophrenia^23, 24^.

There is evidence that *Gria1* ^*−/−*^ mice show behavioural changes that mimic negative symptoms, such as anhedonia, in schizophrenia. Ruling out potential confounds, however, has proved difficult. For example, there are reports of reduced social behaviour^18, 19, 25^, but while these deficits may reflect an aspect of social anhedonia, it is possible that these tests are confounded by deficits in spatial habituation^14, 20, 26^. Similarly, it is possible that other demonstrations of emotional blunting in *Gria1* ^−/−^ mice^17, 27^ were caused by impaired short-term habituation resulting in knockout mice failing to express the normal behavioural response.

The role of GluA1 containing AMPA receptors in anhedonia has also been examined in relation to consumption of palatable foods^18, 25, 28^. While it remains to be seen whether palatability responses to foods provide a measure of anhedonia that is pertinent to the negative symptoms of schizophrenia^29^, specific measures of consummatory behavior in response to palatable foods can be used to gauge hedonic value in animals (see ref. 30 for a review). Previous studies examining sucrose consumption in *Gria1* ^−/−^ mice, however, have examined the quantity of intake and have provided mixed results. Bannerman, *et al*.^28^ found a reduction in 5% glucose intake, whereas Maksimovic, *et al*.^25^ found increased intake of 8% sucrose, and Barkus, *et al*.^18^ found similar levels of consumption of 8% sucrose in wild-type (WT) and GluA1 deficient mice, and the preference for sucrose over water did not differ between groups. The different findings may reflect a number of procedural differences between the studies.

A problem with using the level of consumption of a palatable food as a measure of palatability is that consumption reflects multiple components of feeding behaviour. For example, the consumption of sucrose follows an inverted U-shaped function as concentration increases^31^. Thus, at low concentrations of sucrose, increases in sweetness result in increased intake, but this peaks at intermediate levels and increases in concentration beyond this point result in reduced intake. We have recently demonstrated that mice consume significantly more of 10% sucrose than 2.5% and 20%^32^. Thus, while the level of consumption may reflect a component of palatability indicating the hedonic value of the food, it can also reflect a satiety response reflecting post-ingestive feedback^33^.

Compared to measures of consumption, the manner in which foods are consumed offers greater insight into their palatability. In studies examining the microstructure of licking behaviour it has been found that rodents make clusters of licks (i.e., a series of licks made in quick succession) that are separated by pauses^32, 34^. In contrast to measures of consumption, the mean number of licks per cluster (mean lick cluster size) increases monotonically as a function of sucrose concentration^32, 34, 35^. Therefore, lick cluster size has been proposed as a measure of palatability that is dissociable from measures of consumption (see ref. 30 for a recent review).

In order to provide a purer test of the role of GluA1 containing AMPA receptors in the processing of hedonic value we measured the palatability of sucrose solutions at a variety of concentrations in *Gria1* ^−/−^ mice using a microstructure analysis of licking behaviour that allowed the measurement of lick cluster sizes (the number of licks in succession in which there is less than 0.5 s between the end of one lick and the start of the next^32, 36^). It was found that palatability responses were substantially reduced in *Gria1* ^−/−^ mice in comparison to WT controls, however, measures of consumption were not consistently affected by GluA1 deletion. Furthermore, flavour conditioning, based on flavour-sucrose learning, was unimpaired, suggesting that the role of GluA1 was limited to palatability responses and did not extend to learning based on other properties of sucrose.

## Results

### Experiment 1: The effect of sucrose concentration on licking during a 10 minute test in hungry mice

**Figure 1:**
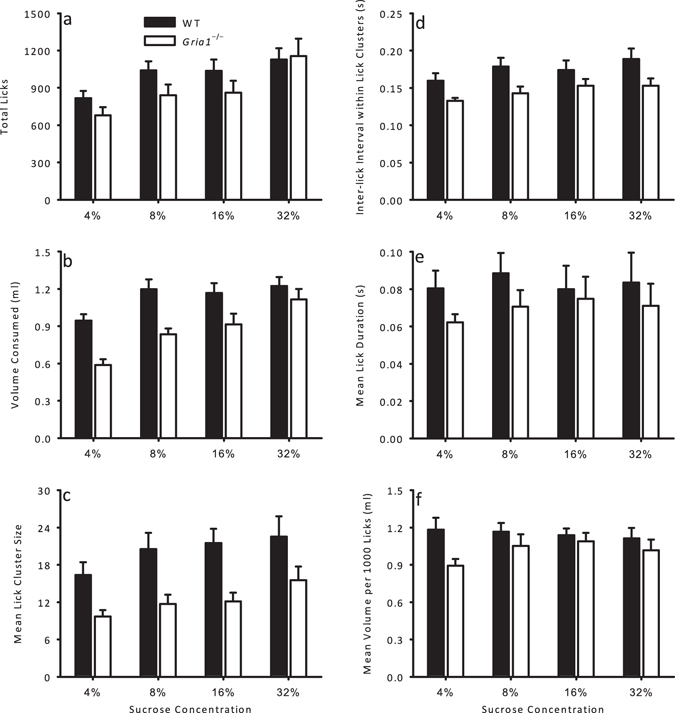
Licking behaviour as a function of sucrose concentration during a short, 10 minute access to sucrose in hungry mice. Six different measures of licking behaviour are given for WT (black bars) and *Gria1* ^−/−^ (white bars) mice for consumption of 4%, 8%, 16%, and 32% sucrose solutions: (**a**) total licks; (**b**) volume consumed (ml); (**c**) mean lick cluster size; (**d**) inter-lick interval within lick clusters (s); (**e**) mean lick duration (s); (**f**) mean volume per 1000 licks (ml). Error bars indicate SEM.

Mice were allowed 10 minutes of access to solutions of 4%, 8%, 16% and 32% sucrose. For both *Gria1* ^−/−^ and WT mice the vast majority of licks were made within clusters of two or more licks (*Gria1* ^−/−^ mean = 97.80% ± 0.45 SEM; WT mean = 99.60% ± 0.08 SEM), however, the proportion of licks made within clusters was significantly higher for WT than *Gria1* ^−/−^ mice, *t*(13) = 3.65, *p* = 0.003. The measures of licking behaviour for *Gria1* ^−/−^ and WT mice are shown in Fig. 1. Summaries of statistical analyses of the main effects and interactions of sucrose concentration and genotype are reported in Table 1. Pairwise comparisons for the significant main effects of sucrose concentration are shown in Table 2. Total licks increased with increasing sucrose concentration similarly for both genotypes (see Fig. 1a). There was a similar increase across concentrations for volume consumed, but for this measure there was an effect of genotype that significantly interacted with sucrose concentration (see Fig. 1b). Simple main effects analysis revealed that *Gria1* ^−/−^ mice consumed less than WT mice of 4%, *p* < 0.001, and 8%, *p* = 0.004, but not 16% or 32%, *p*-values > 0.05. Lick cluster sizes (the number of successive licks with less than 0.5 s between the end of one lick and the start of the next^32, 36^) were significantly lower for 4% sucrose compared to all other concentrations (see Fig. 1c). *Gria1* ^−/−^ mice, however, showed significantly lower lick cluster sizes than WT mice, and the effect of genotype did not interact with sucrose concentration. WT mice showed longer inter-lick intervals within lick clusters than *Gria1* ^−/−^ mice (see Fig. 1d). *Gria1* ^−/−^ mice showed similar mean lick durations and volume per 1000 licks to WT mice across all sucrose concentrations (see Fig. 1e–f).

**Table 1:** Results of mixed-model ANOVAs of concentration (4%, 8%, 16%, or 32%) by genotype (WT or *Gria1* ^−/−^), showing main effects of concentration and genotype, and the concentration x genotype interaction, for six measures of consumption behaviour in Experiments 1 and 2.

Measure	Experiment 1: 10 minute exposure			Experiment 2: 1 hour exposure		
	Concentration (df_1_ = 3, df_2_ = 39)	Genotype (df_1_ = 1, df_2_ = 13)	Interaction (df_1_ = 3, df_2_ = 39)	Concentration (df_1_ = 3, df_2_ = 90)	Genotype (df_1_ = 1, df_2_ = 30)	Interaction (df_1_ = 3, df_2_ = 90)
Total Licks	*F* = 16.8	*F* = 1.18	*F* = 1.77	*F* = 28.6	*F* = 1.30	*F* = 0.64
	*p* < 0.001***	*p* = 0.30	*p* = 0.17	*p* < 0.001***	*p* = 0.26	*p* = 0.52
Volume Consumed	*F* = 27.2	*F* = 10.6	*F* = 3.47	*F* = 63.2	*F* = 0.19	*F* = 0.52
	*p* < 0.001***	*p* = 0.006**	*p* = 0.025*	*p* < 0.001***	*p* = 0.67	*p* = 0.54
Mean Lick Cluster Size	*F* = 9.25	*F* = 9.47	*F* = 0.68	*F* = 14.2	*F* = 34.3	*F* = 9.70
	*p* < 0.001***	*p* = 0.009**	*p* = 0.57	*p* < 0.001***	*p* < 0.001***	*p* < 0.001***
Inter-lick Interval within Lick Clusters	*F* = 3.20	*F* = 8.46	*F* = 0.38	*F* = 0.75	*F* = 4.81	*F* = 0.92
	*p* = 0.034*	*p* = 0.012*	*p* = 0.77	*p* = 0.48	*p* = 0.036*	*p* = 0.40
Mean Lick Duration	*F* = 0.37	*F* = 1.27	*F* = 0.28	*F* = 2.51	*F* = 1.38	*F* = 0.93
	*p* = 0.77	*p* = 0.28	*p* = 0.84	*p* = 0.096	*p* = 0.25	*p* = 0.39
Mean Volume per 1000 Licks	*F* = 0.86	*F* = 2.54	*F* = 1.84	*F* = 5.57	*F* = 0.77	*F* = 0.63
	*p* = 0.47	*p* = 0.14	*p* = 0.16	*p* = 0.008**	*p* = 0.39	*p* = 0.52

Relevant degrees of freedom are given in the table headings (df_1_ = between-groups degrees of freedom; df_2_ = error degrees of freedom). Asterisks denote significance at various levels: *p < 0.05; **p < 0.01; ***p < 0.001.

**Table 2:** Pairwise comparisons of the effect of sucrose concentration on licking behaviour in Experiments 1 and 2.

Pairwise Comparison	Experiment 1: 10 minute exposure			Experiment 2: 1 hour exposure		
	Total Licks	Mean Lick Cluster Size	Inter-lick Interval within Lick Clusters	Total Licks	Volume Consumed	Mean Volume per 1000 Licks
4% vs 8%	*p* = 0.004**	*p* = 0.017*	*p* > 0.05	*p* > 0.05	*p* > 0.05	*p* > 0.05
4% vs 16%	*p* = 0.013*	*p* = 0.036*	*p* > 0.05	*p* < 0.001***	*p* < 0.001***	*p* = 0.014*
4% vs 32%	*p* < 0.001***	*p* = 0.007**	*p* > 0.05	*p* < 0.001***	*p* < 0.001***	*p* > 0.05
8% vs 16%	*p* > 0.05	*p* > 0.05	*p* > 0.05	*p* < 0.001***	*p* < 0.001***	*p* > 0.05
8% vs 32%	*p* = 0.002**	*p* > 0.05	*p* > 0.05	*p* < 0.001***	*p* < 0.001***	*p* > 0.05
16% vs 32%	*p* = 0.027*	*p* > 0.05	*p* > 0.05	*p* < 0.001***	*p* < 0.001***	*p* > 0.05

Holm-Bonferroni-adjusted pairwise comparisons of the significant main effects of sucrose concentration that were identified in Table 1 to have occurred in the absence of a sucrose concentration x genotype interaction. Asterisks denote significance at various levels: *p < 0.05; **p < 0.01; ***p < 0.001.

### Licking behaviour during the first five minutes

**Figure 2:**
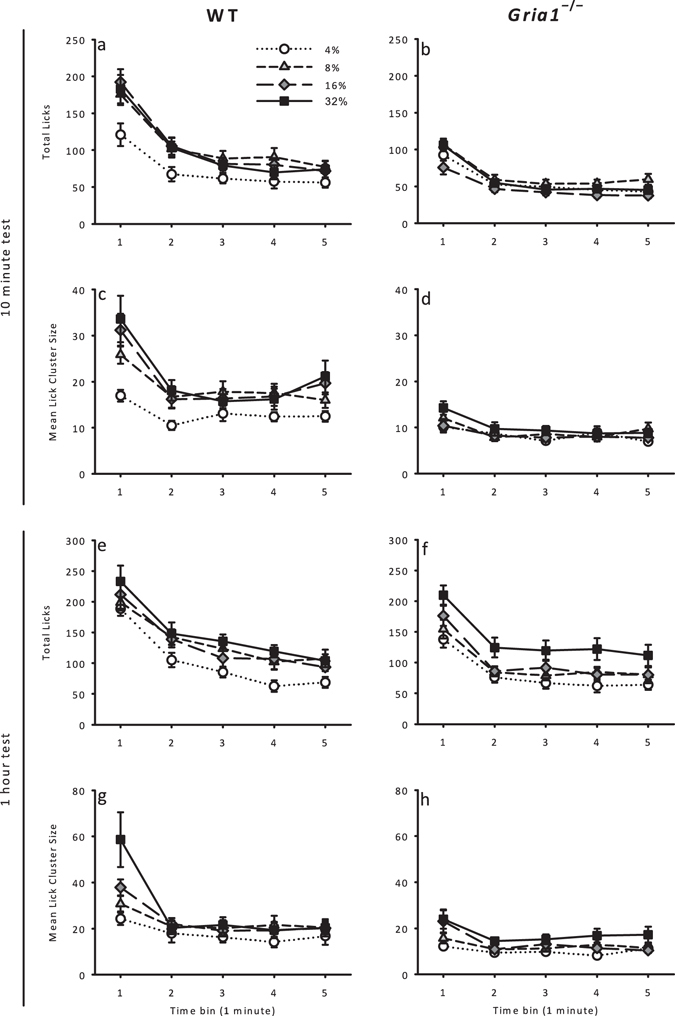
Initial lick rates and lick cluster sizes across the first five minutes of access to sucrose in one-minute time bins in hungry mice. Panels (a–d) show licking behaviour in Experiment 1 (10 minute test) and panels (e–h) show licking behaviour in Experiment 2 (1 hour test). Panels (a,b,e and f) show total licks. Panels (c,d,g and h) show mean lick cluster size. Error bars indicate ± SEM.

Initial lick rates have been suggested to reflect the palatability of the consumed food^37, 38^. Therefore, in order to assess initial lick rate we analysed licking behaviour restricted to the first five minutes of each session, in one-minute time bins. The data for the total licks and mean lick cluster size are shown in Fig. 2 (panels a-d). The number of licks changed with sucrose concentration*, F*(3, 39) = 15.2, *p* < 0.001, and showed a general decrease over bins, *F*(4, 52) = 151, *p* < 0.001 (see Fig. 2a,b). There was a significant bin x genotype interaction, *F*(4, 52) = 4.97, *p* = 0.002. Further analysis of the significant bin x genotype interaction showed that WT mice made a greater number of licks than *Gria1* ^−/−^ mice in bin 2, *F*(1, 13) = 9.22, *p* = 0.010, but not in any other bins, *F*-values < 4.0, *p*-values > 0.05. The effect of bin was significant for WT mice, *F*(4, 24) = 60.7, *p* < 0.001, and for *Gria1* ^−/−^ mice, *F*(4, 28) = 110, *p* < 0.001. All other main effects and interactions were non-significant, *F*-values < 3.5, *p*-values > 0.08.

Mean lick cluster size increased as a function of sucrose concentration, *F*(3, 39) = 9.46, *p* < 0.001 (see Fig. 2c,d). *Gria1* ^−/−^ mice made smaller lick cluster sizes than WT mice, *F*(1, 13) = 10.4, *p* = 0.007, however, this effect interacted with bin, *F*(4, 52) = 10.5, *p* < 0.001. Furthermore, there was a significant concentration x bin x genotype interaction, *F*(12, 156) = 3.82, *p* = 0.035. To analyse the significant three-way interaction, separate genotype x bin ANOVAs were conducted for each sucrose concentration. For the 4%, 8%, and 16% sucrose concentrations, there were significant main effects of bin, *F*-values > 6.4, *p*-values < 0.006, and genotype, *F*-values > 9.0, *p*-values < 0.011, but no significant bin x genotype interactions, *F*-values < 1.8, *p*-values > 0.19. For 32% sucrose, there was a significant bin x genotype interaction, *F*(4, 52) = 9.46, *p* = 0.005. Further analysis of this interaction showed that WT mice had larger lick cluster sizes than *Gria1* ^−/−^ mice in bin 1, *F*(1, 13) = 8.46, *p* = 0.012, but not in any other bins, *F*-values < 3.5, *p*-values > 0.08. Additionally, there was an effect of bin for WT mice, *F*(4, 24) = 14.6, *p* = 0.006, and for *Gria1* ^−/−^ mice, *F*(4, 28) = 7.50, *p* = 0.008.

### Experiment 2: The effect of sucrose concentration on licking during a 1 hour test in hungry mice

**Figure 3:**
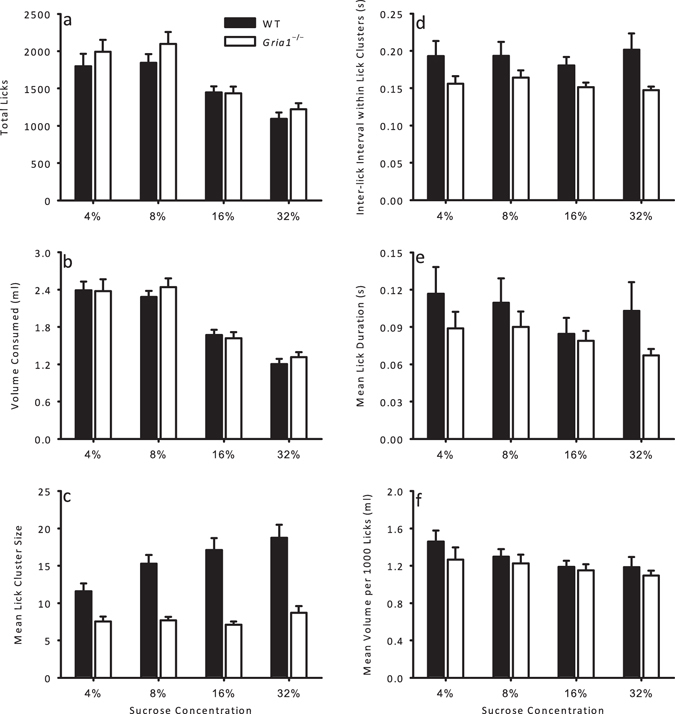
Licking behaviour as a function of sucrose concentration during a long, 1 hour access to sucrose in hungry mice. Six different measures of licking behaviour are given for WT (black bars) and *Gria1* ^−/−^ (white bars) mice for consumption of 4%, 8%, 16%, and 32% sucrose solutions: (**a**) total licks; (**b**) volume consumed (ml); (**c**) mean lick cluster size; (**d**) inter-lick interval within lick clusters (s); (**e**) mean lick duration (s); (**f**) mean volume per 1000 licks (ml). Error bars indicate SEM.

In order to assess consumption over a longer period, in which licking may be affected by satiety, mice were allowed 1 hour of access to solutions of 4%, 8%, 16% and 32% sucrose. For both *Gria1* ^−/−^ and WT mice the vast majority of licks were made within clusters of two or more licks (*Gria1* ^−/−^ mean = 96.70% ± 0.36 SEM; WT mean = 99.02% ± 0.27 SEM), however, the proportion of licks made within clusters was significantly higher for WT than *Gria1* ^−/−^ mice, *t*(30) = 5.10, *p* < 0.001. The measures of licking behaviour for *Gria1* ^−/−^ and WT mice are shown in Fig. 3. Summaries of the main effects and interactions for these analyses can be seen in Table 1. Pairwise comparisons for the significant main effects of sucrose concentration are shown in Table 2. The total licks and volume consumed decreased with increasing sucrose concentration similarly for both genotypes, however there was little difference between 4% and 8% (see Fig. 3a,b). There was a significant genotype by sucrose concentration interaction for lick cluster sizes (see Fig. 3c, and Table 1 for the statistic). Simple main effects analysis revealed that WT mice had lick cluster sizes that increased monotonically with increasing sucrose concentration, *F*(3, 45) = 14.6, *p* < 0.001, whereas *Gria1* ^−/−^ mice did not differ with changing sucrose concentration, *F*(3, 45) = 2.48, *p* = 0.12. WT mice had higher lick cluster sizes than *Gria1* ^−/−^ mice for all sucrose concentrations, *F*-values > 10.9, *p*-values < 0.003.

WT mice showed higher inter-lick intervals within lick clusters than *Gria1* ^−/−^ mice (see Fig. 3d). This measure did not differ with sucrose concentration for either genotype. The mean lick duration and mean volume per 1000 licks were similar between genotypes (see Fig. 3e-f). Mean volume per 1000 licks decreased across concentrations, but mean lick duration did not.

### Licking behaviour during the first five minutes

As for Experiment 1 we assessed initial lick rates by restricting analysis of licking to the first five minutes of the test, measured in one-minute time bins. For the measures of total licks and mean lick cluster size, data can be seen for the first five minutes of exposure, in one-minute time bins, in Fig. 2 (panels e-h). Total licks was, on the whole, greater for higher concentrations than for lower concentrations*, F*(3, 90) = 4.66, *p* = 0.004, and decreased over bins, *F*(4, 120) = 131*, p* < 0.001 (see Fig. 2e,f). *Gria1* ^−/−^ mice made significantly fewer licks than WT mice, *F*(1, 30) = 26.1, *p* < 0.001. There was a significant concentration x bin x genotype interaction, *F*(12, 360) = 2.71, *p* = 0.002. To analyse the significant three-way interaction, separate genotype x bin ANOVAs were conducted for each sucrose concentration. For 4% sucrose, there was no significant bin x genotype interaction, *F* < 1, *p* > 0.4. For 8% sucrose, there was a significant bin x genotype interaction, *F*(4, 120) = 4.58, *p* = 0.002. Further analysis of this interaction showed that WT mice made more licks than *Gria1* ^−/−^ mice in bins 1–4, *F*-values > 7.2, *p*-values < 0.012, but not in bin 5, *F*(1, 30) = 2.60, *p* = 0.12. For 16% and 32% sucrose, there were significant bin x genotype interactions, *F*-values > 7.8, *p*-values < 0.001. Further analyses of these interactions showed that WT mice made more licks than *Gria1* ^−/−^ mice in all bins, *F*-values > 4.6, *p*-values < 0.04.

Mean lick cluster size increased as a function of sucrose concentration, *F*(3, 90) = 6.58, *p* < 0.001 (see Fig. 2g,h). Lick cluster size decreased over bins, *F*(4, 120) = 43.1, *p* < 0.001, and *Gria1* ^−/−^ mice made smaller lick clusters than WT mice, *F*(1, 30) = 37.9, *p* < 0.001. There was a significant concentration x bin x genotype interaction, *F*(12, 360) = 2.31, *p* = 0.045. To further analyse the significant three-way interaction, separate genotype x bin ANOVAs were conducted for each sucrose concentration. For 4% sucrose, there were significant main effects of bin, *F*(4, 120) = 7.28, *p* < 0.001, and genotype, *F*(1, 30) = 18.2, *p* < 0.001, and a significant bin x genotype interaction, *F*(4, 120) = 2.80, *p* = 0.029. Further analysis of this interaction showed that WT mice had larger lick cluster sizes than *Gria1* ^−/−^ mice on bins 1, 3, 4, and 5, *F*-values > 4.2, *p*-values < 0.048, but not on bin 2, *F*(1, 30) = 1.91, *p* = 0.18. For 8%, 16%, and 32% sucrose, there were significant main effects of bin, *F*-values > 11.1, *p*-values < 0.001, and genotype, *F*-values > 15.9, *p*-values < 0.001, and significant bin x genotype interactions, *F*-values > 2.63, *p*-values < 0.038. Further analyses of these interactions showed that WT mice had larger lick cluster sizes than *Gria1* ^−/−^ mice in all bins, *F*-values > 8.3, *p*-values < 0.008.

### Experiment 3: Water and sucrose consumption over a 1 hour test in thirsty mice

**Figure 4:**
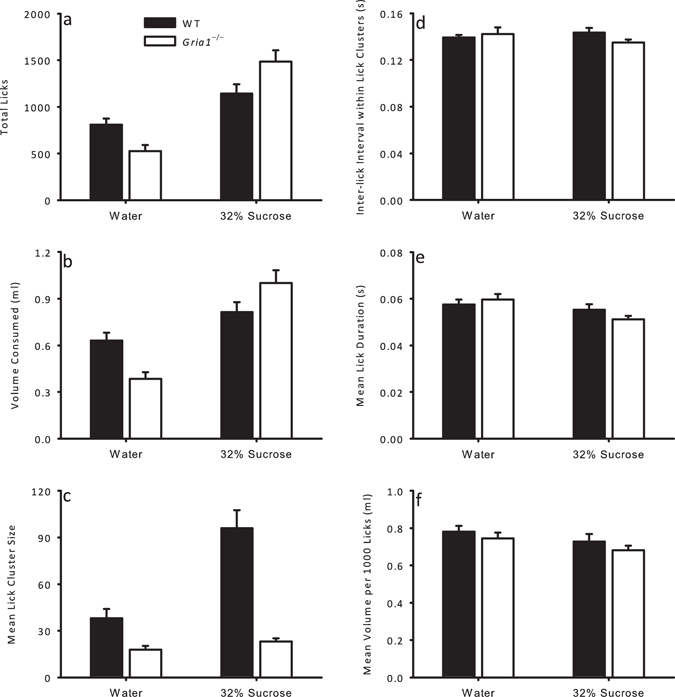
Licking behaviour during a 1 hour access to either water or 32% sucrose in thirsty mice. Six different measures of licking behaviour are given for WT (black bars) and *Gria1* ^−/−^ (white bars) mice for consumption of water and 32% sucrose solution: (**a**) total licks; (**b**) volume consumed (ml); (**c**) mean lick cluster size; (**d**) inter-lick interval within lick clusters (s); (**e**) mean lick duration (s); (**f**) mean volume per 1000 licks (ml). Error bars indicate SEM.

In order to assess the generality of the GluA1-dependent effects in Experiments 1 and 2 mice were tested with either water or 32% sucrose, but now under restricted access to water, such that mice were motivated to drink by thirst. For both *Gria1* ^−/−^ and WT mice the vast majority of licks were made within clusters of two or more licks (*Gria1* ^−/−^ mean = 98.06% ± 0.41 SEM; WT mean = 98.74% ± 0.35 SEM) and there was no significant difference between genotypes, *t*(36) = 1.27, *p* = 0.21. The measures of licking behaviour for *Gria1* ^−/−^ and WT mice are shown in Fig. 4. Summaries of the main effects and interactions for these analyses can be seen in Table 3. There was a significant genotype x group interaction for both the total number of licks and volume consumed (see Fig. 4a,b and Table 3 for the statistics). Simple main effects analysis showed that the total number of licks and consumption for water was greater in WT mice compared to *Gria1* ^−/−^ mice (*total licks*: *F*(1, 34) = 4.83, *p* = 0.035; *volume consumed*: *F*(1, 34) = 7.99, *p* = 0.008). The reverse was true for 32% sucrose, with *Gria1* ^−/−^ mice making more licks and consuming more than WT mice (*total licks*: *F*(1, 34) = 7.07, *p* = 0.012; *volume consumed*: *F*(1, 34) = 4.67, *p* = 0.038). For both genotypes total number of licks and consumption was greater for 32% sucrose than for water (WT mice: *total licks*: *F*(1, 34) = 6.93, *p* = 0.013, *volume consumed*: *F*(1, 34) = 4.73, *p* = 0.037; *Gria1* ^−/−^ mice: *total licks*: *F*(1, 34) = 52.3, *p* < 0.001, *volume consumed*: *F*(1, 34) = 48.0, *p* < 0.001).

**Table 3:** Results of univariate ANOVAs of group (water or 32% sucrose) by genotype (WT or *Gria1* ^−/−^), showing main effects of group and genotype, and the group x genotype interaction, for six measures of consumption behaviour in Experiment 3.

Measure	Experiment 3: 1 hour exposure (thirst motivated)		
	Group (df_1_ = 1, df_2_ = 34)	Genotype (df_1_ = 1, df_2_ = 34)	Interaction (df_1_ = 1, df_2_ = 34)
Total Licks	*F* = 49.8	*F* = 0.11	*F* = 11.8
	*p* < 0.001***	*p =* 0.75	*p* = 0.002**
Volume Consumed	*F* = 42.5	*F* = 0.22	*F* = 12.4
	*p* < 0.001***	*p* = 0.64	*p* = 0.001**
Mean Lick Cluster Size	*F* = 20.6	*F* = 44.8	*F* = 14.3
	*p* < 0.001***	*p* < 0.001***	*p* = 0.001**
Inter-lick Interval within Lick Clusters	*F* = 0.23	*F* = 0.50	*F* = 1.58
	*p* = 0.64	*p* = 0.48	*p* = 0.22
Mean Lick Duration	*F* = 6.09	*F* = 0.21	*F* = 2.06
	*p* = 0.019*	*p* = 0.65	*p* = 0.16
Mean Volume per 1000 Licks	*F* = 3.23	*F* = 1.53	*F* = 0.03
	*p* = 0.081	*p* = 0.22	*p* = 0.86

Relevant degrees of freedom are given in the table headings (df_1_ = between-groups degrees of freedom; df_2_ = error degrees of freedom). Asterisks denote significance at various levels: *p < 0.05; **p < 0.01; ***p < 0.001.

For lick cluster sizes genotype interacted with group (see Fig. 4c and Table 3 for statistic) such that whereas WT mice made larger lick clusters for 32% sucrose than for water, *F*(1, 34) = 36.6, *p* < 0.001, *Gria1* ^−/−^ mice did not, *F* < 1, *p* = 0.60. Lick cluster sizes were smaller in *Gria1* ^−/−^ mice than WT mice for both water, *F*(1, 34) = 4.23, *p* = 0.048, and 32% sucrose, *F*(1, 34) = 54.8, *p* < 0.001. For all other measures (inter-lick intervals within lick clusters, mean lick duration, and volume consumed per 1000 licks), behaviour was similar for WT and *Gria1* ^−/−^ mice regardless of whether water or sucrose was being consumed (see Fig. 4d–f).

### Experiment 4: Flavour conditioning with limited (1 session) training in hungry mice

#### Training

In order to assess learning based on the properties of sucrose mice were exposed to a flavour paired with a high concentration of sucrose and another flavour paired with a low concentration of sucrose. Flavour conditioning is demonstrated by increased intake of the flavour previously paired with a high concentration of sucrose compared to the other flavour when both flavours are paired with the same concentration of sucrose^36^. Mice were allowed a single 10 minute period of access to a flavour mixed with 32% sucrose (CS+) and a 10 minute period of access to another flavour mixed with 4% sucrose (CS−). The total licks, volume consumed and mean lick cluster size during training are reported in Table 4. Total licks and volume consumed were higher to 32% sucrose solution than to 4% (*total licks: F*(1, 28) = 87.5, *p* < 0.001; *volume consumed: F*(1, 28) = 134, *p* < 0.001). *Gria1* ^−/−^ mice made a greater number of total licks compared to WT mice, *F*(1, 28) = 6.68, *p* = 0.015, but the effect of genotype for volume consumed was not significant, *F*(1, 28) = 1.09, *p* = 0.31. The effect of genotype did not significantly interact with sucrose concentration for either measure (*total licks*: *F*(1, 28) = 2.97, *p* = 0.096; *volume consumed*: *F*(1, 28) = 3.46, *p* = 0.074).

**Table 4:** Flavour conditioning training data for Experiments 4 and 5.

Measure	Experiment 4: 1 session training				Experiment 5: 8 sessions training			
	WT		*Gria1* ^−/−^		WT		*Gria1* ^−/−^	
	4%	32%	4%	32%	4%	32%	4%	32%
Total Licks	316 (76)	790 (81)	428 (57)	1116 (81)	307 (64)	785 (99)	266 (34)	1021 (127)
Volume Consumed (ml)	0.36 (0.09)	0.93 (0.10)	0.35 (0.06)	1.13 (0.05)	0.40 (0.06)	0.94 (0.07)	0.22 (0.06)	0.96 (0.08)
Mean Lick Cluster Size	9.2 (1.3)	17.3 (2.2)	7.4 (0.7)	11.3 (1.0)	10.2 (1.5)	15.1 (3.4)	6.7 (0.5)	11.6 (1.6)

Mean (SEM) total licks, volume consumed, and lick cluster size for WT and *Gria1* ^−/−^ mice for 4% and 32% sucrose solutions during training for Experiments 4 and 5.

Mean lick cluster size was larger for 32% sucrose than for 4% in both genotypes, although this difference was much greater in the WT mice. There was a significant sucrose concentration x genotype interaction, *F*(1, 28) = 4.58, *p* = 0.041. Further analysis of this significant interaction showed that mean lick cluster size was greater for 32% sucrose than for 4% for WT mice, *F*(1, 28) = 33.6, *p* < 0.001, and *Gria1* ^−/−^ mice, *F*(1, 28) = 7.68, *p* = 0.010. WT mice, however, showed a greater mean lick cluster size than *Gria1* ^−/−^ mice for 32% sucrose, *F*(1, 28) = 6.51, *p* = 0.016, but not for 4%, *F*(1, 28) = 1.68, *p* = 0.21.

#### Test

**Figure 5:**
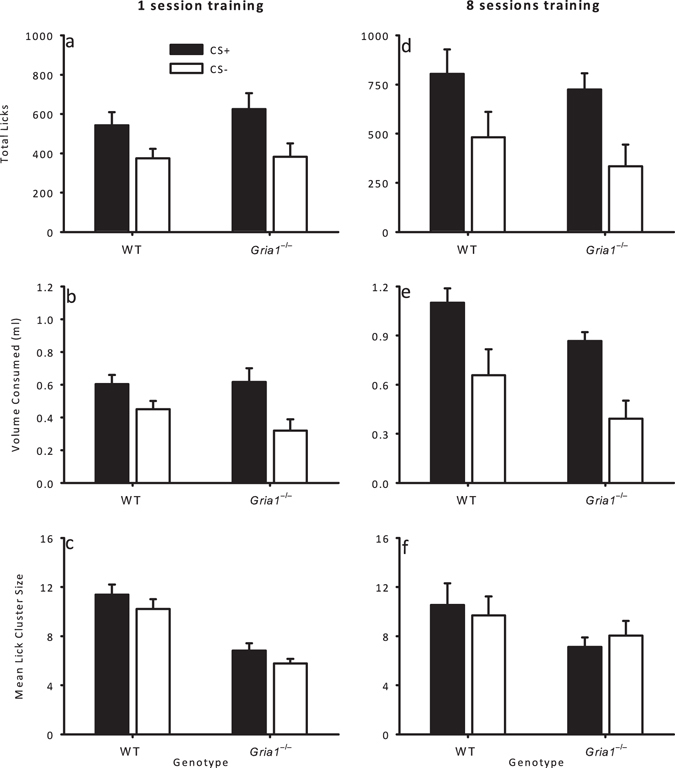
Flavour conditioning as a consequence of limited (one session) and extended (eight sessions) training. Measures of responding to the CS+ flavour and the CS− flavour are shown by black and white bars respectively. The measures of licking behaviour for limited training (Experiment 4) are shown in the left column and those for extended training (Experiment 5) are shown on the right. Panels (**a**) and (**d**) show total licks, (**b**) and (**e**) show volume consumed (ml), and (**c**) and (**f**) show mean lick cluster size. Error bars indicate SEM.

Twenty four hours after training mice were allowed access to the CS+ and CS− flavours (10 minutes per flavour), but now both flavours were mixed with 4% sucrose. Greater consumption of the CS+ would indicate conditioning to that flavour. Total licks, volume consumed, and mean lick cluster size during the test session are shown in Fig. 5 (panels a-c). Total licks and volume consumed were higher for the CS+, which had previously been paired with 32% sucrose, than to the CS−, which had been paired with 4% sucrose *(total licks: F*(1, 28) = 13.4, *p* = 0.001; *volume consumed*: *F*(1, 28) = 22.6, *p* < 0.001). There were no overall differences between genotypes (*total licks* and *volume consumed*: *F*-values < 1, *p*-values > 0.4) and no significant interactions between sucrose concentration and genotype (*total licks* and *volume consumed*: *F*-values < 2.3, *p*-values > 0.1).

Mean lick cluster size was larger overall for WT mice than for *Gria1* ^−/−^ mice, *F*(1, 28) = 29.2, *p* < 0.001, and the mean lick cluster size for the CS+ was higher than that for the CS−, *F*(1, 28) = 6.11, *p* = 0.020. The stimulus x genotype interaction was not significant, *F*(1, 28) = 0.02, *p* = 0.90.

### Experiment 5: Flavour conditioning with extended (8 sessions) training in hungry mice

#### Training

Experiment 4 failed to show a difference in flavour conditioning between *Gria1* ^−/−^ and WT mice. It is possible that the single exposure to the flavours in Experiment 4 did not provide sufficient training to allow a difference between genotypes to emerge. Therefore, in Experiment 5 naïve mice received extended training with the flavours prior to the test of learning. Mice were allowed eight 10 minute periods of access to a flavour mixed with 32% sucrose (CS+) and eight 10 minute periods of access to another flavour mixed with 4% sucrose (CS−), with one period of access to each flavour per day. Total licks, volume consumed, and mean lick cluster size across all eight training sessions are reported in Table 4. Total licks and volume consumed were higher for the 32% sucrose than to 4% (*total licks*: *F*(1, 11) = 71.8, *p* < 0.001; *volume consumed*: *F*(1, 11) = 77.0, *p* < 0.001). Both measures were similar between genotypes, *F*-values < 1.5, *p*-values > 0.2, and there was no significant interaction of sucrose concentration and genotype, *F*-values < 3.7, *p*-values > 0.08.

Mean lick cluster size was larger for 32% sucrose than for 4% in both genotypes, *F*(1, 11) = 13.4, *p* = 0.004. WT mice had larger lick cluster sizes than *Gria1* ^−/−^ mice, but this effect was not significant, *F*(1, 11) = 1.68, *p* = 0.22. There was no significant interaction of sucrose concentration and genotype, *F* < 1, *p* > 0.9.

### Test

Twenty four hours after training mice were allowed access to the CS+ and CS− flavours (10 minutes per flavour), but now both flavours were mixed with 4% sucrose. Total licks, volume consumed, and mean lick cluster size during the test session are shown in Fig. 5 (panels d-f). Total licks and volume consumed were higher for the CS+, which had previously been paired with 32% sucrose, than for the CS−, which had been paired with 4% sucrose (*total licks*: *F*(1, 11) = 11.6, *p* = 0.006; *volume consumed*: *F*(1, 11) = 12.7, *p* = 0.004). The genotypes did not differ in total licks, *F* < 1, *p* > 0.3, but *Gria1* ^−/−^ mice consumed less than WT mice, *F*(1, 11) = 6.70, *p* = 0.025. There was no sucrose concentration x genotype interaction for either measure, *F*-values < 1, *p*-values > 0.7.

Mean lick cluster size for WT mice was slightly larger for the CS+ than the CS− flavour, but this was not the case for *Gria1* ^−/−^ mice. However, there was no sucrose concentration by genotype interaction, *F*(1, 11) = 1.28, *p* = 0.28, and the main effects of sucrose concentration, *F* < 1, *p* > 0.9, and genotype, *F*(1, 11) = 1.81, *p* = 0.21, were not significant.

## Discussion

The experiments reported here examined the effect of GluA1 deletion on palatability responses to sucrose solutions. Palatability was assessed using lick cluster size, rather than just overall levels of consumption. It was found that GluA1 deletion drastically reduced the number of licks in a cluster for a range of sucrose concentrations, regardless of whether mice were motivated to consume due to hunger or thirst. In contrast, hunger-motivated consumption, when measured over an hour-long period, was normal in *Gria1* ^−/−^ mice. Furthermore, *Gria1* ^−/−^ mice were clearly sensitive to the difference between sucrose concentrations and showed normal flavour conditioning, based on experience of flavours paired with different sucrose concentrations. The findings demonstrate that GluA1 deletion impaired the hedonic response to a food, but left satiety responses and sucrose based learning intact.

*Gria1* ^−/−^ mice, when hungry, showed a reduction in lick cluster size both when tested over a 10 minute and an hour long period. Furthermore, when initial consumption during the first five minutes was assessed, *Gria1* ^−/−^ mice showed an immediate reduction in lick cluster size. Therefore, the reduction was not due to overly rapid adaptation to sucrose, but instead reflects a reduction in the automatic response to sucrose. The reduced lick cluster size is in contrast to other aspects of licking behaviour that were unaffected by GluA1 deletion. Specifically *Gria1* ^−/−^ mice showed normal lick duration and volume consumed per lick. Given this, it is unlikely that the reduction in lick cluster size reflects a general deficit in licking. However, the inter-lick interval within lick clusters was shorter for *Gria1* ^−/−^ mice than WT mice, when mice were hungry. The shorter inter-lick intervals within lick clusters should, all other things being equal, lead to larger lick cluster sizes due to fewer inter-lick intervals exceeding the 0.5 s inter-cluster interval criterion. Indeed, it has been argued that some reductions in lick cluster size are due to longer inter-lick intervals within lick clusters^39^. Therefore, it seems unlikely that the shorter inter-lick intervals were the cause of the reduced lick cluster sizes in *Gria1* ^−/−^ mice. Furthermore, in addition to the data reported here, we also analysed lick clusters using 0.25 and 1 s inter-cluster interval criteria. GluA1 deletion reduced lick cluster sizes regardless of the inter-cluster interval criterion. Importantly, when mice were thirsty, *Gria1* ^−/−^ mice showed reduced lick cluster sizes in the absence of any effect on the inter-lick interval within lick clusters. It is unclear why *Gria1* ^−/−^ mice had shorter inter-lick intervals than WT mice under food restriction but not water restriction, but differences in motivational levels to drink might be a contributing factor to this difference.

Consistent with other studies of lick cluster size in rodents^32, 34, 35^, lick cluster size increased monotonically as a function of sucrose concentration in WT mice. This effect was evident when mean lick cluster sizes were calculated over a variety of durations of access to sucrose. This was in contrast to the relationship between measures of consumption and sucrose concentration. When measured over 10 minutes, total licks and volume consumed tended to increase as a function of sucrose concentration, but when measured over one hour, total licks and volume consumed tended to decrease with increasing concentrations beyond 8%. These results are consistent with the suggestion that initial lick rates reflect the palatability of a solution^37, 38^, but licking over longer periods is affected by satiety responses that lead to reductions in consumption of high sucrose concentrations. Thus, consumption over time is optimal for intermediate sucrose concentrations. The dissociable effects of sucrose concentration on lick cluster size and consumption demonstrate that cluster size is more directly linked to palatability.

When measured over an hour-long period, measures of consumption did not differ between hungry *Gria1* ^−/−^ and WT mice. *Gria1* ^−/−^ mice showed the same decrease in total licks and volume consumed with increasing sucrose concentration as WT mice, demonstrating that they were sensitive to the differences in the concentration. However, rates of consumption were lower in *Gria1* ^−/−^ mice when measured over 10 minutes. As previously mentioned, initial rates of consumption likely reflect palatability^37, 38^. Therefore, the reduced consumption in *Gria1* ^−/−^ mice in the 10-minute exposure is consistent with the findings of reduced lick cluster size, indicating a decrease in palatability. These findings were also evident in the analyses of the initial five-minutes of access to sucrose.

When mice were thirsty GluA1 deletion reduced consumption of water, but, in contrast, increased consumption of sucrose over the period of an hour. This is in contrast to the normal levels of consumption of sucrose evident in *Gria1* ^−/−^ mice over the same time period when hungry. It is possible that GluA1 deletion reduced thirst such that water consumption was lower, but this does not account for the increase in consumption of sucrose in *Gria1* ^−/−^ mice when thirsty. Although it is not clear why GluA1 deletion had opposite effects on the consumption of water and sucrose when thirsty, the fact that lick cluster sizes were reduced for both water and sucrose in *Gria1* ^−/−^ mice provides a further demonstration that the deficit in lick cluster size was independent of overall levels of licking and consumption.

The fact that GluA1 deletion reduced the mean lick cluster size for water, which may be considered to be a relatively neutral stimulus in terms of palatability, may suggest that *Gria1* ^−/−^ mice have a general deficit in making lick clusters such that rapid licking cannot be sustained. There is evidence, however, that water activates acid-sensing taste receptor cells, suggesting that it is not neutral and may have some hedonic value when drinking is motivated by thirst^40^. Therefore, the reduced lick cluster size for water in *Gria1* ^−/−^ mice may still simply reflect impaired hedonic responding. While it cannot be ruled out entirely that *Gria1* ^−/−^ mice have a motor deficit that results in reduced lick cluster size, the fact that there were not consistent changes to other aspects of licking behavior, as mentioned previously, makes this possibility appear unlikely. More generally, it is not clear whether GluA1 deletion reduced the perceived palatability of foods, or whether instead it simply impaired the behavioural expression of palatability. At present it is not possible to dissociate these two accounts.

Despite the reduction in mean lick cluster size, *Gria1* ^−/−^ mice showed successful flavour learning. In the test phase of the conditioning procedure mice were presented with flavours, both mixed with 4% sucrose, but one flavour had previously been paired with 32% and one with 4% sucrose in the training phase. Therefore, a difference in consumption in the test phase reflects learning based on the prior exposure^36^. *Gria1* ^−/−^ and WT mice showed a similar conditioning effect with greater consumption of the flavour previously paired with 32% than the flavour paired with 4%. This was true when mice received one training exposure with each flavour and when mice received eight training exposures with each flavour. Therefore, it is unlikely that the lack of effect of genotype is due to either a floor or ceiling effect. Importantly, the normal learning in *Gria1* ^−/−^ mice demonstrates that they are able to discriminate between different sucrose concentrations. Furthermore, the fact that *Gria1* ^−/−^ mice consumed more of a flavour previously paired with 32% sucrose than a flavour previously paired with 4% sucrose provides further evidence that motivation for sucrose is not impaired.

The results of the present experiments provide the first clear evidence that GluA1 containing AMPA receptors play a role in hedonic responses, and that GluA1 deletion models some of the negative symptoms of disorders such as schizophrenia. Crucially, the results now highlight the need for greater understanding of the role of glutamatergic signalling in palatability and hedonia generally. It has been suggested that schizophrenia may reflect an altered interaction between the glutamatergic and dopaminergic systems^1^. Consistent with this proposal, GluA1 deletion has been found to reduce the rate of extracellular dopamine clearance in the striatum^19^, resulting in hyperdopaminergia, suggesting that this may be a cause of the altered, schizophrenia-like behaviours in *Gria1* ^−/−^ mice. Hyperdopaminergia per se, however, does not result in the pattern of performance seen with *Gria1* ^−/−^ mice. Thus, hyperdopaminergia, as modeled by the dopamine transporter knockout mouse, actually results in increased lick cluster sizes^41^. Hedonia has also been linked to endogenous opioid neurotransmission^42, 43^, raising the possibility that GluA1 deletion may affect this system. Indeed, GluA1 has been implicated in aspects of opiate-dependent learning^44^, and plays a role in regulating synaptic plasticity in areas in which opioid neurotransmission has been implicated in aspects of hedonia^45–48^. Direct manipulation of opioid receptors on palatability as measured by lick cluster sizes, however, have failed to find an effect^49, 50^. Given the lack of clarity, there is now a need for better understanding of the primary neurobiological causes of hedonic reactions and the effect of glutamatergic dysfunction on those processes.

It is possible that GluA1 deletion impairs responding based on hedonic value by affecting the function of the basolateral amygdala. The basolateral amygdala contains neurons that fire in response to palatability that also modulate firing in the gustatory cortex^51, 52^, and the basolateral amygdala is important for encoding the sensory-specific incentive motivational properties of food rewards^53^. GluA1 deletion mimics the effects of lesions of the basolateral amygdala on sensory-specific encoding^15, 16, 54^, such that behavior is dependent on stimulus-response associations rather than response-outcome associations^55^. The present results may build on this analysis by further demonstrating that GluA1 is necessary for the automatic, unconditioned behavioural response to food based on its sensory-specific qualities, as well as conditioned responding caused by the associative retrieval of sensory-specific qualities of food.

In conclusion, the results presented here show that GluA1 is necessary for specific consummatory behaviours that relate to the palatability of food. These results provide a new insight into the neural basis of palatability and responding based on hedonic value.

## Methods

### Subjects

Subjects were *Gria1* ^−/−^ mice and WT age-matched littermates, bred in the Life Sciences Support Unit at Durham University (see ref. 8 for details of genetic construction, breeding and subsequent genotyping). The mice were originally derived from the 129S2svHsd and C57BL/6 J/OlaHsd strains, and have subsequently been backcrossed onto the C57BL/6 J line. The majority of mice had previously been used in unrelated appetitive magazine approach studies, and half of the mice in Experiment 2 had previously been used in Experiment 4. The mice in Experiment 3 had previously been used in an unrelated appetitive magazine approach study and an unrelated study involving the consumption of flavoured sucrose solutions. Mice were caged in groups of one to nine in a temperature controlled housing room on a 12-hour light-dark cycle (light period: 8am to 8 pm). The mice were 11–30 weeks old at the start of testing, with weights ranging from 14.6–33.4 g. For experiments 1, 2, 4, and 5, prior to the start of testing, weights were reduced by restricting access to food and mice were maintained at 85% of their free-feeding weights throughout the experiment. These mice had ad libitum access to water in their home cages. For experiment 3, mice had ad libitum access to food in their home cages, but were given only one hour of access to water per day in their home cage (in addition to any liquids consumed during the experimental procedure). All procedures were in accordance with the United Kingdom Animals (Scientific Procedures) Act 1986 and were approved by the UK Home Office under project license number PPL 70/7785.

### Apparatus

A set of eight identical operant chambers (interior dimensions: 21.6 × 17.8 × 12.7 cm; ENV-307W, Med Associates, Inc., Fairfax, VT, USA), enclosed in sound-attenuating cubicles (ENV-022V, Med Associates) were used. The operant chambers were controlled by Med-PC IV software (Med Associates). The side walls were made from aluminium, and the front and back walls and the ceiling were made from clear Perspex. The chamber floors each comprised a grid of 24 stainless steel rods (0.32 cm diameter), spaced 0.79 cm apart and running perpendicular to the front of the chamber (ENV-307W-GFW, Med Associates). Retractable sippers (ENV-352AW, Med Associates) and a small hole in one wall of each chamber allowed graduated pipettes to be extended into, and retracted from, the chambers. The graduated pipette (10:0.1 ml) allowed measurement of consumption by comparing the volumes before and after testing. Contact lickometer controllers (ENV-250, Med Associates) allowed contacts between the mice and the graduated pipettes to be recorded at a resolution of 0.01 s. A fan (ENV-025F, Med Associates) was located within each of the sound-attenuating cubicles and was turned on during sessions. Sucrose solutions were made weight/volume with commercially available sucrose in distilled water. Flavours used were cherry, grape, orange and apple Kool Aid (0.05% w/v, Kraft Foods USA, Rye Brook, NY, USA).

### Procedure

#### Experiment 1: The effect of sucrose concentration on licking during a 10 minute test in hungry mice

*Gria1* ^−/−^ (4 female, 4 male) and WT mice (3 female, 4 male) were allowed to consume 4%, 8%, 16%, and 32% sucrose solutions on twelve sessions, with one session per day, arranged in three blocks of four sessions such that each sucrose concentration was consumed once per block. The order in which the concentrations were presented was counterbalanced as far as possible within each genotype within each 4-session block, such that half of the *Gria1* ^−/−^ mice and approximately half of the WT mice received the two low concentrations (4% and 8%) in the first two sessions and the remaining mice received the two high concentrations (16% and 32%) with the order of each concentration counterbalanced across mice. Then, in the third and fourth sessions, mice received the two remaining concentrations in an order approximately counterbalanced with respect to the order of the initial two concentrations. Sessions lasted 15 minutes, with the pipette extended into the chamber for the final 10 minutes.

#### Experiment 2: The effect of sucrose concentration on licking during a 1 hour test in hungry mice

*Gria1* ^−/−^ (8 female, 8 male) and WT mice (8 female, 8 male) received 8 sessions, two per concentration, of exposure to sucrose. Sessions lasted 65 minutes, with the pipette extended into the chamber for the final 60 minutes. All other details were identical to Experiment 1.

#### Experiment 3: Water and sucrose consumption over a 1 hour test in thirsty mice

*Gria1* ^−/−^ (12 female, 6 male) and WT mice (8 female, 12 male) that were under mild water deprivation were allowed to consume water for four sessions (data not shown). Mice were then split into two groups, with 6 female and 3 male *Gria1* ^−/−^ and 4 female and 6 male WT mice per group, matched for mean lick cluster size within genotype and sex. For another four sessions, one group continued to receive water while the other group had access to 32% sucrose solution instead. Each session lasted 65 minutes, with the pipette extended into the chamber for the final 60 minutes.

#### Experiment 4: Flavour conditioning with limited (1 session) training

*Gria1* ^−/−^ (8 female, 7 male) and WT mice (7 female, 8 male) received a single training session, consisting of two trials with an inter-trial interval of approximately 10 minutes. On one trial, mice were allowed to drink 4% sucrose solution paired with one flavour (the CS−), and on the other trial they could drink 32% sucrose solution paired with another flavour (the CS+). The order in which these trials were presented was approximately counterbalanced within genotype. Approximately half of the mice were trained with cherry and grape Kool Aid, and the other mice with orange and apple Kool Aid. The allocation of flavours to CS+ and CS− training was counterbalanced as far as possible within genotype. Each trial lasted 15 minutes, with the pipette extended into the chamber for the final 10 minutes. Twenty-four hours following the training session, mice were given a single test session using the same procedure as during training except that mice received both the CS+ and CS− flavours paired with 4% sucrose solution. The order of presentation of flavours during the test session was counterbalanced as far as possible with respect to the previous counterbalancing.

#### Experiment 5: Flavour conditioning with extended (8 sessions) training

*Gria1* ^−/−^ (3 female, 3 male) and WT mice (3 female, 4 male) received eight sessions of training with one CS+ trial and one CS− trial (approximately 10-min ITI) per session. The order of trial types (CS+, CS−) alternated across consecutive sessions. All other procedures were the same as Experiment 4.

### Data Analysis

Multiple aspects of licking behaviour were recorded and analysed: total number of licks, volume consumed, mean lick cluster size, mean inter-lick interval (lick onset to subsequent lick onset) within lick clusters, mean lick duration, and mean volume consumed per 1000 licks. A lick cluster was defined as a series of two or more licks made with less than 0.5 s between the end of one lick and the start of the next.

All data were analysed using multifactorial ANOVA. Interactions were analysed with simple main effects analysis using the pooled error term from the original ANOVA or separate ANOVAs for repeated measures with more than two levels. Where sphericity of within-subjects variables could not be assumed, a Greenhouse-Geisser correction was applied. Post-hoc analyses used the Holm-Bonferroni correction to control for multiple comparisons.

For those experiments with adequate sample-sizes (Experiments 2 and 4), all analyses were initially conducted including sex as a between-subjects factor. Male mice showed larger lick cluster sizes than female mice in Experiment 2 (overall and during the first five minutes) and in the test stage of Experiment 4. Males also made a higher number of licks than females in Experiment 2, but this was not observed in other situations. The only time sex interacted with any other factors was for the total number of licks made during the training stage of Experiment 4. Here, female *Gria1* ^−/−^ mice made more licks than female WT mice (but this was not true for males), and female WT mice made fewer licks than male WT mice (but this was not true for *Gria1* ^−/−^ mice). Overall, therefore, sex had a very limited effect on the measures of licking behaviour and thus this factor was excluded from the main analyses for ease of exposition.

### Data availability statement

The datasets generated during and/or analysed during the current study are available from the corresponding author on reasonable request.
